# Isolated Resistance Training Programs to Improve Peripheral Muscle Function in Outpatients with Chronic Obstructive Pulmonary Diseases: A Systematic Review

**DOI:** 10.3390/healthcare9101397

**Published:** 2021-10-19

**Authors:** Simone Pancera, Nicola F. Lopomo, Luca N. C. Bianchi, Paolo Pedersini, Jorge H. Villafañe

**Affiliations:** 1 IRCCS Fondazione Don Carlo Gnocchi, 20148 Milan, Italy; lubianchi@dongnocchi.it (L.N.C.B.); pedersini93@gmail.com (P.P.); mail@villafane.it (J.H.V.); 2 Department of Information Engineering, University of Brescia, Via Branze 38, 25123 Brescia, Italy; nicola.lopomo@unibs.it

**Keywords:** chronic obstructive pulmonary disease, pulmonary rehabilitation, resistance training, muscle strength, systematic review

## Abstract

This systematic review aims to establish which isolated resistance training (RT) programs have been used in outpatients with chronic obstructive pulmonary disease (COPD) and their impact on all aspects of peripheral skeletal muscle function. Electronic databases were systematically searched up to June 2021. The eligibility criteria were: (1) randomized controlled trials investigating the effects of supervised and isolated RT programs in outpatients with COPD and (2) RT programs lasting 8–12 weeks, (3) including at least one outcome measure related to trainable muscle characteristics. Initially, 6576 studies were identified, whereas 15 trials met the inclusion criteria. All the included trials reported that isolated RT improved both upper and lower limbs’ maximal strength. Muscle endurance and power also increased after RT but received less attention in the analysis. Furthermore, few studies assessed the effect of RT on muscle mass and cross-sectional area, reporting only limited improvement. Isolated RT programs carried out 2–3 days a week for 8–12 weeks improved skeletal muscle function in individuals with COPD. The RT program should be specifically focused to the trainable muscle characteristic to be improved. For this reason, we further encourage the introduction of a detailed assessment of muscle function and structure during the pulmonary rehabilitation practice.

## 1. Introduction

Peripheral muscle dysfunction in patients with chronic obstructive pulmonary disease (COPD) results in a combination of intrinsic modifications—including muscle fiber shift, changes in capillarization, mitochondrial disorder, and oxidative damage—and functional limitations [1]. The most clinically relevant consequences of these alterations are loss of muscle mass, muscle weakness, and an inability to sustain or even perform exercise [2]. Furthermore, all these factors could lead individuals with COPD to an increased use of healthcare and a deterioration in their quality of life, thus aggravating the overall socio-economic burden of this disease [3,4].

Resistance training (RT) refers to the exercise performed by local muscle groups against body weight or external resistance and represents a key component in the comprehensive pulmonary rehabilitation (PR) program addressing patients with COPD [5]. Moreover, RT is not limited to muscle strength exercises but refers to further trainable muscle characteristics, such as muscle power and local muscle endurance [6]. Indeed, there is evidence that all these muscle characteristics contribute to enhancing the overall function of peripheral muscles in individuals with COPD, inducing structural and metabolic adaptations and improving the patient’s functional exercise capacity [7,8].

However, the RT programs available for treating patients diagnosed with COPD are characterized by a great variability; hence, it is difficult to properly assess their short- and long-term effectiveness [9]. In fact, even though these protocols are usually adapted according to the patients’ existing condition and clinical status, the recommendations for the assessment and treatment of muscle dysfunction in individuals with COPD are frequently poor of proper indications for clinical practice. In addition, there is increasing evidence that specific aspects of muscle function have currently received less attention with respect to muscle strength [10]. Therefore, specific RT programs and methods targeting these emerging perspectives on muscle function should be preferred to a generalized approach that may be unable to improve the compromised function of the patients [11,12]. In this perspective, practical aspects of RT regarding the trainable muscle characteristics to be improved, the target muscle groups, and the choice of appropriate load and volume should be further elucidated in order to increase the rehabilitation tools available to therapists during PR programs for individuals with COPD [13].

Moreover, though RT is an established component of PR and has been included in almost 70% of PR programs in Europe, with a minimum recommended length of 24 sessions, strength outcomes are not yet considered as one of the most important measures in the clinical evaluation of patients with COPD [1,14]. For this reason, the assessment of peripheral muscles function is poorly integrated in the clinical routine in most cases [13,15]. Therefore, there is an increasing need to identify appropriate standard and complementary evaluations of peripheral muscles function in order to better define the progression and effects of RT programs in patients with COPD [16].

Some earlier reviews have explored the effects of RT on respiratory function and exercise capacity in individuals with COPD [17,18,19], whereas other reviews focused on the effects of exercise training on muscle strength [20,21,22]. However, these reviews often included studies on combined RT and endurance training (ET) programs, reviewed multiple design studies, or even reported outcomes that are limited to a single aspect of peripheral muscle function. Therefore, considering the heterogeneity of the interventions currently adopted to improve peripheral muscle function and the need for addressing important clinical issues concerning present and emerging RT methods used in patients diagnosed with COPD, a systematic review was conducted in order to clarify these aspects directly related to the PR practice. 

More specifically, this systematic review aimed to establish which isolated RT programs have been used in outpatients with COPD and their impact on peripheral muscle strength, local muscle endurance, and muscle power. Additionally, the effects of these treatments on the cross-sectional area (CSA) and fat-free mass (FFM) of the muscles are presented. An overview of the methods used to assess these muscle characteristics is also provided.

## 2. Methods

In accordance with guidelines [23], the protocol for this systematic review was registered in the International Prospective Register of Systematic Reviews (PROSPERO) on 14 February 2020, under identification number CRD42020168650.

### 2.1. Search Strategy

The primary search was conducted for English, French-, Spanish-, or Portuguese-language studies published up to June 2020 in the following electronic databases: MEDLINE (PubMed), The Cochrane Central Register of Controlled Trials (CENTRAL), Web of Science, Embase, and Scopus. The secondary search was carried out for reference lists, focusing on all the included papers and reviews performed on the same topic. The search was re-run in June 2021 to retrieve new studies suitable for inclusion in this systematic review. The complete search strategy used in the main databases is provided in the Table S1 (Supplementary Materials).

The literature search and sifting process were conducted by two separate reviewers (SP, JHV), applying previously determined inclusion criteria. A third neutral investigator (NFL) was questioned when conflicts arose between the reviewers.

### 2.2. Eligibility Criteria

This systematic review included randomized controlled trials investigating the effects of supervised and isolated (i.e., performed as the main and only intervention) RT programs on any trainable muscle characteristic (i.e., muscle strength, muscle power, and local muscular endurance) in outpatients diagnosed with COPD (Global Initiative for Obstructive Lung Disease, GOLD stage I–IV) [24]. Primary outcomes included any objective measure of muscle strength endurance or power with no restrictions regarding the assessment method used (e.g., one repetition maximum, dynamometry, force plates). Secondary outcomes included measures of muscle CSA and FFM. The duration of the RT program could vary between 8 and 12 weeks (≥24 sessions), and the exercises could be carried out using external resistance (free weights, weight machines, or elastic bands) or body weight training. Intervention in the control group could involve ET, combined RT and ET, breathing exercises, education, or usual care. Studies were excluded if they used home-based RT programs, passive training methods, nutritional, or pharmacological supplementation as the main intervention. Studies that enrolled healthy subjects as sole controls were also excluded.

### 2.3. Data Extraction

The percentage of change from baseline for each outcome measure of the included studies related to muscle strength, endurance, and power was extracted and reported in the results section. If not available, these percentages were calculated by the reviewers using pre- and post-training values for each outcome measure. Information about number and size of groups, duration, type of intervention, and protocol of RT (i.e., frequency, volume, intensity, load, and progression) were also extracted from each included study

### 2.4. Quality Assessment

The methodological quality of the included studies was assessed using the Physiotherapy Evidence Base Database (PEDro) scale that was reported to be a valid measure of the methodological quality for clinical trials [25]. Included studies were rated with a minimum score of 0 and a maximum score of 10 points [26] and were considered to be of “good” to “excellent” quality when scoring ≥6 points, while studies scoring ≤5 points were defined as “low” to “fair” quality [27].

## 3. Results

### 3.1. Study Selection

**Figure 1:**
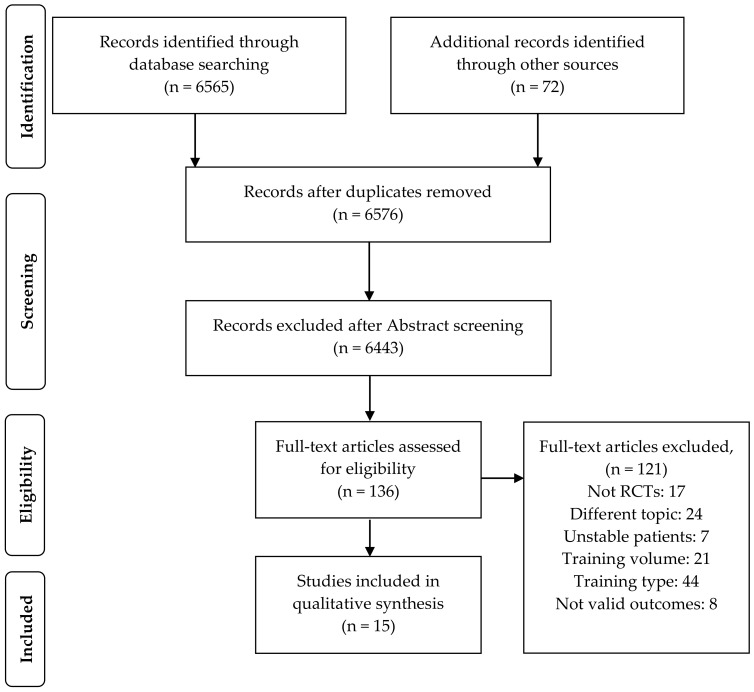
Flow diagram of the study selection. RCTs, randomized controlled trials.

A total of 6576 studies were initially identified through database searching, of which 136 full-text articles were assessed for eligibility. Overall, 121 studies were excluded because they did not meet the inclusion criteria, as shown in Figure 1, whereas 15 trials published between February 1992 and May 2021 were finally included in the systematic review.

### 3.2. Quality Rating

The quality of included studies was rated ≤ 5 points in seven trials [28,29,30,31,32,33,34] and ≥6 points in eight other studies [35,36,37,38,39,40,41,42]. The sample size of the included studies varied between 12 and 48 subjects and was calculated a priori in 7 out of 15 trials [29,33,34,36,37,41,42]. The attrition rate was ≤ 10% in four studies [28,35,36,40], between 11% and 30% in eight studies [29,30,31,33,34,37,38,41], and ≥30% in three studies [32,39,42]. The quality assessment of the included studies is detailed in Table 1.

### 3.3. Study Characteristics

The total number of patients with COPD in the 15 included studies was 493 (mean age, 63 years; mean percentage of predicted forced expiratory volume in 1 second, 48%), with a percentage of female subjects of 25%. The length of the training programs ranged from 24 and 36 sessions, with a training frequency of two or three times weekly, and each session lasted between 60 and 90 min (Table 2). 

The initial training load prescribed was between 50% and 85% of the one-repetition maximum or based on the maximal load that could be lifted between 15 and 30 times. The subsequent training load was established by repeating the maximal test [28,29,30,31,32,33,38], by using the Borg scale [36], or with increases of predetermined loads [35], or percentages of the maximal load achieved by patients [37,39,42]. The number of days before the recalculation of the workload, if specified, varied between 2 and 50 days. The included trials prescribed two to seven sets for each exercise, with 5–30 repetitions and 1–3 min of rest. The gym equipment used in the studies was represented by free weights, weight machines, pulleys, and elastic tubing or bands. 

There were 13 studies that adopted RT programs for both the upper and lower limbs [28,29,31,32,33,34,36,37,38,39,40,41,42], while two studies exercised only the lower limbs [30,35]. In two trials, the training program was designed to enhance the peripheral muscle endurance [36,42], imposing a moderate-fast velocity of contraction, whereas two studies focused on the muscle power, giving emphasis to the explosive movement performed during the concentric phase of the exercise [30,35]. Most of the remaining trials did not report a specific velocity of contraction or adopted a 1:1 velocity of contraction in the concentric and eccentric phases. 

The control groups performed no intervention, education, or breathing exercise in 6 out of 15 studies [28,30,35,36,38,40]. Three studies compared RT with ET [31,34,39], whereas one trial made a comparison between RT and light-intensity training or a combination of both [29]. The remaining five studies compared RT carried out with weights by using different elastic resistances [32,33,37,41] or even involving one or two limbs at a time [42].

### 3.4. Outcome Measures

**Figure 2:**
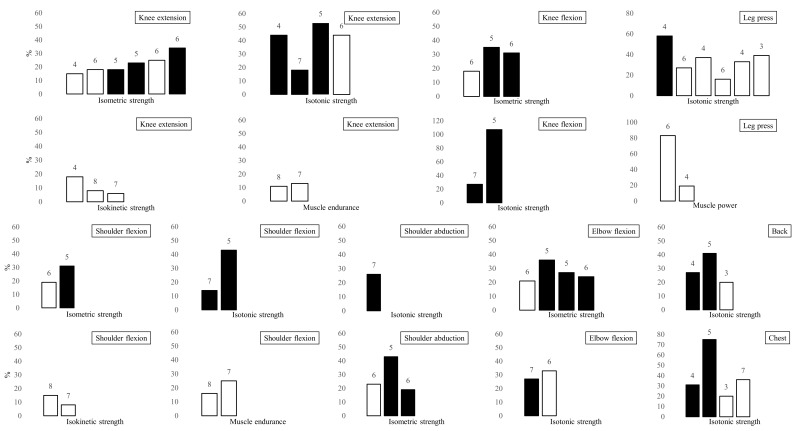
Harvest plots of the effect of isolated RT on peripheral muscle function in outpatients with COPD. Changes for the various outcome measures are expressed as percentage of baseline. For studies that adopted more than one modality of RT (e.g., conventional RT or RT with elastic bands), the highest increase was reported. Numbers above the bars refer to the quality score of the studies (from 0 to 10). The length of the training program is indicated by black bars (36 sessions) or white bars (24 sessions). Combined exercises for chest muscles (i.e., chest press and butterfly) and back muscles (i.e., lat pull) are grouped in the “chest” and “back” graphs, respectively.

The main outcome measures of muscle function are summarized in Table S2 (Supplementary Materials) and Figure 2. The maximal isometric strength of the knee extensors was evaluated in six studies, where an increase between 15% and 34% was found after RT [30,32,33,37,38,39]. Five trials assessed the maximal isotonic strength of the knee extensors, reporting improvements from 18% to 53% [28,29,31,38,41]. The maximal isokinetic strength of the knee extensors was measured in four trials and changed between 5% and 18% from baseline [28,30,36,42]. In addition, three studies assessed the muscle endurance of the knee extensors, and two of them reported an 11% increase after a RT program focused on muscle endurance [36,42]. Five trials measured the maximal strength of the knee flexors, finding an improvement between 18% and 35% and between 27% and 107% from baseline in isometric [32,37,39] and isotonic [31,41] conditions, respectively. The combined leg press exercise was tested in six studies that reported an increase between 16% and 58% [29,30,34,35,38,40]. In addition, the overall muscle power of the lower limbs was assessed in three studies: one of these found an improvement of 83% in the rate of force development [35], whereas two studies measured the power output and found a 19% increase and no change, respectively [30,40]. 

The maximal isometric strength of the elbow flexors was measured in four studies that reported an improvement between 21% and 36% [32,33,37,39]. Two studies investigated the maximal isotonic strength of the elbow flexors, but only two of them reported an increase between 23% and 33% after the RT program [38,41]. The maximal isometric strength of the shoulder flexors showed an improvement between 19% and 43% in two studies [32,37], while the maximal isokinetic strength of the shoulder flexors was measured in other two studies, showing an increase between 8% and 15% from baseline [36,42]. The same trials assessed the muscle endurance of the shoulder flexors, finding a 16% or a 21% improvement [36,42], respectively. The maximal strength during combined exercises for chest muscles (i.e., chest press and butterfly) were evaluated in four studies that reported an increase between 20% and 75% from baseline, respectively [29,31,34,40], whereas three trials reported an improvement between 20% and 27% in the combined exercises for back muscles (i.e., lat pull) [29,31,34]. The handgrip strength was found to have increased by 18% [38] in one study and did not change in two other studies that measured it [29,39]. 

Concerning secondary outcomes, one trial showed a 4% increase of the CSA of the quadriceps after RT, measured via magnetic resonance imaging [30]. Two studies assessed the FFM using bioimpedance analysis (BIA) and one study using dual-energy X-ray absorptiometry (DEXA). These trials found no change or 4% of increase after RT in the first case [29,41] and a 2% of increase in the second [37].

## 4. Discussion

The main objective of this systematic review was to explore the literature about the available isolated RT programs used for the treatment of outpatients with COPD and their impact on the patient’s peripheral muscle function. All 15 included trials reported a positive effect of isolated RT programs on maximal isometric, isotonic, and isokinetic muscle strength of both the upper and lower limbs and, to a limited extent, also on local muscle endurance and muscle power after 8 to 12 weeks of training. A small number of studies also showed a positive impact of RT on muscle CSA and FFM in patients with COPD. In general, exercise interventions showed a large variability with regard to the program design, the prescription and progression of the training load, and the volume of training recommended. The reported outcome measures were also heterogeneous for both the choice of the muscle group and the muscle function being assessed. The included studies reported an overall quality ranging from “fair” to “good”.

### 4.1. RT Program Design

This systematic review found that RT programs for outpatients with COPD are usually of 24 or 36 sessions, 2–3 days a week. However, since individuals with COPD suffer from early fatigability, their level of conditioning and recovery ability should be taken into account when prescribing the optimal RT frequency [43]. In any case, a certain degree of muscle fatigue is suggested in order to induce a functional adaptation to training in patients with COPD [44].

The training intensity should also be target specific; starting, for example, with initial lighter loads (45–50% of one repetition maximum) or higher number of repetitions (15–30) may represent a suitable strategy for deconditioned or frail individuals, such as individuals with COPD [12]. Then, the initial load should be progressively increased up to 80%—or more—in order to focus the training effect on maximal muscle strength or maintained to a lower extent in order to improve, for example, local muscle endurance [36,42]. In fact, most of the studies included in this systematic review chose an initial load within this wide range, and with few exceptions, all the trials adopted a load-increasing strategy to intensify the exercise over time. However, highly variable progression strategies were found with regards to the timing and magnitude of the increase; therefore, it is difficult to speculate on the proper progression strategy for patients with COPD so far.

### 4.2. Impact of RT on Trainable Muscle Characteristics

Despite the large variability among the interventions, all the studies included in this systematic review reported an improvement in maximal muscle strength testing after RT, with more limited and lower-quality evidences for the upper compared to the lower limbs. Handgrip strength was the only exception to this trend, probably because it represents the strength of the forearm muscles rather than the overall muscle strength of the upper limbs; thus, specific training is required to improve it [45]. Moreover, in contrast with the findings of this systematic review, the maximal strength of the knee extensors did not change in 17% of the studies included in a previous review, thus suggesting that patients with COPD may respond in a different manner to similar RT programs [22]. Nevertheless, contrary to this systematic review, those authors reviewed an elevated number of studies with multiple design and different RT modalities.

The local muscle endurance—usually reduced in individuals with COPD [46]—improved in both the upper and lower limbs in particular when a high number of repetitions and a minimum recovery were used [36,42]. Furthermore, as found in two studies included in the systematic review, there is a strong correlation between change in isokinetic muscle endurance and change in treadmill endurance [28] or change in the number of capillaries per muscle fiber [42] after RT. Therefore, these results empower the previously suggested idea that RT may induce similar benefits on physical and metabolic function compared with aerobic training [7,47]. Moreover, considering the lower cardiopulmonary stress induced by RT [48], a training regime focused on the improvement of the local muscle endurance might be suitable for individuals with COPD. In particular, performing exercises using a single limb at a time could increase the participation of those patients who are severely limited by dyspnea or are unable to sustain prolonged or high-intensity ET [42].

This systematic review also highlighted the improvement of lower-limb muscle power after RT in patients with COPD, particularly when the emphasis was on the explosivity of the exercise (i.e., explosive concentric phase and slow eccentric phase), and high loads (80 to 90% of 1RM) were adopted [30,35]. However, using relatively low loads (50 to 70% of 1RM) produced slighter effects on muscle power, unless RT was combined with ET, as shown in one study included in this systematic review [40]. In contrast, no data were available for muscle power of the upper limbs, probably due to the difficulty in measuring it. Since muscle power has been reported to be reduced by 30% in patients with COPD, there is increasing support for its inclusion in RT programs during PR [49]. In addition, this muscle characteristic may be associated with light-intensity activities and functional performance (e.g., gait speed), as seen in previous studies and confirmed by the findings of this systematic review [30,50].

### 4.3. Training Modes

Consistent with previous work [20,21], the studies included in this systematic review reported improvements in maximal muscle strength of the upper and lower limbs when RT was compared to no or light intervention. In addition, although limited to the lower limbs, a greater enhancement in local muscle endurance and power was also observed for patients with COPD undergoing RT compared to non-exercising controls [28,30,36]. The comparison between isolated RT and ET programs, on the other hand, gave contrasting results, as reported in a previous review on this topic [47]. However, the findings of this systematic review suggest that the reason for such conflicting results may lie in the difference of intensity prescribed in these studies [31,39]. Surprisingly, in one study, RT failed to produce further gains in the isometric maximal strength of the upper limbs when compared to ET [39]. Nevertheless, these authors additionally exercised the endurance group with arm cranking, which might have produced improvements in the maximal strength of the upper limbs. Furthermore, this systematic review reported a similar increase in maximal muscle strength when RT was isolated or combined with ET [29,31]. However, one of these studies suggests that enhancing peripheral muscle strength with an appropriate RT program may optimize the performance of tasks related to functional exercise capacity in patients with COPD [29].

Finally, the studies included in this systematic review reported similar gains in maximal muscle strength after RT with weight machines or elastic bands; thus, the superiority of one modality over the other was not established [32,33,37,41]. In addition, with evidence limited to one study [42], training with one rather than two limbs at a time did not appear to have a different impact on maximal muscle strength and endurance of the upper and lower limbs but only induced less exertional dyspnea in the former case, as previously mentioned.

### 4.4. Methods to Assess Muscle Function in Clinical Practice

The quadriceps were the reference muscle group for assessing muscle function using isometric strength testing, particularly with the hand-held dynamometer or, in a few studies, with the computerized dynamometer. The hand-held dynamometer is obviously well suited for the clinical setting; nevertheless, it involves a risk of over or underestimation of muscle gains [51]. On the other hand, the isokinetic evaluation showed good reliability and accuracy in patients with COPD, but it is costly and time consuming for clinical practice [45]. Moreover, the isokinetic dynamometer may be useful for the evaluation of local muscle endurance despite the differences in measurement protocols between the included studies [28,36,42].

When the maximal muscle strength was assessed dynamically, the preferred method was to test the isotonic one-repetition maximum using the same equipment as during the training. In line with a previous review, the isotonic strength was found to be more responsive to RT compared with the other maximal strength outcomes, probably due to the “familiarization” with the testing device [22].

In this systematic review, as in the current literature, the evaluation of muscle power received less attention among individuals with COPD although it may provide important clinical value [50]. In addition, even when muscle power was evaluated, different devices and protocols were used between the included studies, which does not allow the comparison of results [30,35,40].

### 4.5. Structural and Systemic Effects of RT

This systematic review found weak evidence that RT was suitable for improving the CSA of the quadriceps; nevertheless, this characteristic would require further attention. Since the CSA of the quadriceps represents an independent predictor of survival, simplified and reliable methods should be widely introduced to assess this muscle characteristic in clinical practice [52].

FFM has also been shown to decline with COPD disease severity [2], and this was evaluated in this systematic review using two different methods, with conflicting results: (1) BIA represents a non-invasive, inexpensive, and rapid methodology; (2) DEXA provides more accurate results but involves logistical difficulties and costs [53]. Therefore, in PR practice, the choice of measurement method is likely to be determined by the availability of resources and equipment [54].

### 4.6. Study Limitations

This review presents potential limitations. (1) A limited number of randomized controlled trials were found that investigated RT in isolation for patients with COPD despite a methodologically accurate research. (2) Many of the included studies had a risk of bias due, for example, to an inadequate sample size or the absence of a power calculation as well as variation in measured outcomes and treatments carried out by the control group. (3) The risk of publication bias is an inherent limitation of any systematic review; the authors tried to limit this risk by searching for unpublished studies or non-English-language studies, but nevertheless, the impact of the publication bias was not calculated.

## 5. Conclusions

This systematic review provides an overview of the isolated RT programs that have been used so far in outpatients with COPD and identifies gaps in the current literature, producing recommendations for future research on this topic. Relying only on high-quality studies, supervised, isolated RT was found to be effective in improving maximal muscle strength of both the upper and lower limbs in outpatients with COPD when carried out 2 to 3 days a week for 8 to 12 weeks, performing three series of 8 to 15 repetitions with loads between 70 and 90% of 1RM. When the objective is instead to improve local muscle endurance, a lower load and a higher number of repetitions (25–30) should be preferred. Conversely, to enhance muscle power, which represents an interesting but poorly explored perspective in clinical practice, emphasis should be placed on the explosivity of the exercise when a high-load (85–90% of 1RM) regime is applied. However, designing a RT program for outpatients with COPD requires a preliminary assessment of each trainable muscle characteristic and a further adjustment of the training prescription according to the required number of repetitions, velocity of contraction, load, and progression necessary to improve the specific aspect of muscle function being trained. Furthermore, to obtain a complete framework of all the aspects of patients’ muscle function, the evaluation of muscle strength, endurance, and power should be integrated with the assessment of structural characteristics, such as muscle CSA and body FFM, following the requirements of the clinical rehabilitation practice. A secondary aspect in RT design, on the other hand, is the choice of the equipment for RT, which should be made on the basis of its availability in the clinical setting and safety for patients since there are no differences between, for example, weights or elastic resistance in terms of gain in muscle strength. Finally, adopting training strategies, such as partitioning the exercising muscle groups, namely exercising using a single limb at a time, might represent an alternative to improve muscle dysfunction for those patients who are particularly limited by exertional dyspnea. However, more studies on this topic are necessary to make recommendations.

## Figures and Tables

**Table 1:** Quality assessment of the included studies.

Study	Randomization	Concealed Allocation	Baseline Comparability	Blind Subjects	Blind Therapists	Blind Assessor	FU	ITT	Between-Group Comparison	Point Estimates and Variability	PEDro Score	Attrition Rate (%)
Clark [28]	1	0	1	0	0	0	1	0	1	1	5/10	0
Dourado [29]	1	0	1	0	0	0	0	0	1	1	4/10	28
Freire [41]	1	1	1	0	0	1	0	1	1	1	7/10	27
Hoff [35]	1	0	1	0	0	0	1	1	1	1	6/10	0
Kongsgaard [30]	1	0	1	0	0	0	0	0	1	1	4/10	28
Nyberg [36]	1	1	1	0	0	1	1	1	1	1	8/10	9
Nyberg [42]	1	1	1	0	0	1	0	1	1	1	7/10	30
Ortega [31]	1	0	1	0	0	0	1	0	1	1	5/10	13
Ramos [37]	1	1	1	1	0	0	0	0	1	1	6/10	24
Silva [32]	1	0	1	0	0	1	0	0	1	1	5/10	32
Silva [33]	1	1	1	0	0	0	0	0	1	1	5/10	27
Simpson [38]	1	0	1	0	0	1	1	0	1	1	6/10	18
Spruit [39]	1	1	1	0	0	1	0	0	1	1	6/10	38
Vonbank [34]	1	0	1	0	0	0	0	0	0	1	3/10	16
Zambom-Ferraresi [40]	1	1	1	0	0	1	1	0	1	1	7/10	10

FU, follow-up; ITT, intention-to-treat analysis.

**Table 2:** Characteristics of the included studies.

Study	Study Groups	Study Intervention	Study Duration	Training Protocol
Clark [28]	Conventional resistance training (CO): 26; Control (CG): 17	CO: 8 exercises (chest press, body squat, squat calf, lat machine, arm curls, leg press, knee extension, knee flexion) with weights. CG: No intervention.	12 weeks	Frequency: 2 d/w Reps: 3 × 10 Phase velocity: NA Rest: NA Load: 70% of 1RM Progression: Every 6 weeks (repeating 1RM test)
Dourado [29]	Conventional resistance training (CO): 11; Low-intensity training (LIT): 13; Combined training (CT): 11	CO: 7 exercises (leg press, leg extension, lat pull down, chest press, seated rowing, triceps pulley, and biceps curl) with weight machines. LIT: 30 min of walking and 30 min of low-intensity CO with free weights, on exercise mats and on parallel bars. CT: 30 min of CO group and 30 min as LIT group.	12 weeks	Frequency: 3 d/w Reps: 3 × 12 (CO); 2 × 8 (CT) Phase velocity: NA Rest: 2 min Load: 50–80% of 1RM Progression: Every 3 weeks (repeating 1RM test)
Freire [41]	Conventional resistance training (CO): 16; Elastic tubing resistance (ER): 18; Elastic bands resistance (EB): 14	CO: 5 exercises (shoulder abduction, elbow flexion, shoulder flexion; knee extension and knee flexion) with weight machines. ER and EB: The same exercise program of CO was carried out with elastic tubing or bands.	12 weeks	Frequency: 3 d/w Reps: 2 × 15 (weeks 1–2); 3 × 15 (weeks 3–6); 3 × 10 (weeks 7–9); 3 × 15 (weeks 10–12) Phase velocity: 2 s Rest: 2 min Load: established with nRM Progression: Each session with the nRM test
Hoff [35]	Conventional resistance training (CO): 6; Control (CG): 6	CO: 1 exercise (leg press). CG: No intervention.	8 weeks	Frequency: 3 d/w Reps: 4 × 5 Phase velocity: Explosive concentric, slow eccentric Rest: 2 min Load: 85–90% of 1RM Progression: 2.5 kg increment when 5 reps were exceeded
Kongsgaard [30]	Conventional resistance training (CO): 6; Control (CG): 7	CO: 3 exercises (leg press, knee extension, knee flexion) with weight machines. CG: Breathing exercise.	12 weeks	Frequency: 2 d/w Reps: 4 × 8 Phase velocity: Explosive concentric Rest: 2–3 min Load: 80% of 1RM Progression: Every week
Nyberg [36]	Elastic bands resistance (EB): 22; Control (CG): 22	EB: 8 exercises (latissimus row, chest press, leg extension, straight arm shoulder flex, leg curl, elbow flexion, leg heel raise, leg step-up). CG: 4 days of education.	8 weeks	Frequency: 3 d/w Reps: 2 × 25 Phase velocity: 1 s Rest: 1 min Load: Established nRM Progression: Every 2 sessions (if Borg scale < 4)
Nyberg [42]	Elastic band single-limb resistance (SEB): 16; Elastic band two-limb resistance (TEB):17	SEB: 7 exercises (knee extension, leg curl, latissimus row, chest press, elbow flexion, shoulder flexion, calf) with a single limb at a time. TEB: As SEB but using both limbs at a time.	8 weeks	Frequency: 3 d/w Reps: 3 × 25–30 Phase velocity: 1 s Rest: 1 min Load: Established nRM Progression: Increased every two sessions by 10% if patients exceeded 30 reps
Ortega [31]	Conventional resistance training (CO): 17; Endurance training (ET): 16; Combined training (CT): 14	CO: 5 exercises (lat pull, butterfly, neck press, leg flexion, leg extension) with gymnastic apparatus. ET: 40 min of cycling at 70% of peak work capacity. CT: 20 min of cycling plus CO.	12 weeks	Frequency: 3 d/w Reps: 4 × 6–8 (CO); 2 × 6–8 (CT) Phase velocity: NA Rest: NA Load: 70–85% of 1RM Progression: Every 2 weeks (repeating 1RM test)
Ramos [37]	Conventional resistance training (CO): 17; Elastic tubing resistance (ER): 17	CO: 5 exercises (knee extension, knee flexion, shoulder abduction, shoulder flexion, elbow flexion) with weight machines. ER: Same exercises as CO group, performed with elastic tubing.	8 weeks	Frequency: 3 d/w Reps: 3 × 10 (CO); 2–7 × maximum in 20 s (ER) Phase velocity: NA Rest: 2 min Load: 60% (week 1) to 80% (week 8) of 1RM Progression: Increased by 4% every four sessions (CO); increased by one set every two sessions (ER)
Silva [32]	Conventional resistance training (CO): 10; Elastic tubing resistance (ER): 9	CO: 5 exercises (knee flexion, knee extension, shoulder flexion, shoulder abduction, elbow flexion) with weight machines. ER: Same exercises as CO group, performed with elastic tubing.	12 weeks	Frequency: 3 d/w Reps: 2 × 15 (weeks 1–3); 3 × 15 (weeks 4–6); 3 × 10 (weeks 7–9); 4 × 6 (weeks 10–12) Phase velocity: 1.8 s Rest: 2 min Load: 15RM Progression: Increased when patients exceeded the nRM
Silva [33]	Conventional resistance training (CO): 11; Elastic resistance (ER): 24	CO: 5 exercises (knee flexion, knee extension, shoulder flexion, shoulder abduction, elbow flexion) with weight machines. ER: Same exercises as CO group, performed with elastics.	12 weeks	Frequency: 3 d/w Reps: 2 × 15 (weeks 1–3); 3 × 15 (weeks 4–6); 3 × 10 (weeks 7–9); 3 × 15 (weeks 10–12) Phase velocity: NA Rest: NA Load: Established nRM Progression: Increased when patients exceeded the nRM
Simpson [38]	Conventional resistance training (CO): 14; Control (CG): 14	CO: 3 exercises with weights using a single limb at a time (arm curl, leg extension, leg press). CG: No intervention.	8 weeks	Frequency: 3 d/w Reps: 3 × 10 Phase velocity: Slow concentric Rest: NA Load: 50% (week 1) to 85% (week 8) of 1RM Progression: Every 6 sessions (repeating 1RM test)
Spruit [39]	Conventional resistance training (CO): 14; Endurance training (ET): 16	CO: 6 exercises (quadriceps, pectorals, triceps brachia, deltoids, biceps brachia, hamstrings) with weight machines. ET: Cycling or walking for 25 min at 75% of peak work or 60% of 6-min walk speed) plus arm cranking (4–9 min).	12 weeks	Frequency: 3 d/w Reps: 3 × 8 Phase velocity: NA Rest: NA Load: 70% of 1RM Progression: Increased by 5% of 1RM every week
Vonbank [34]	Conventional resistance training (CO): 12; Endurance training (ET): 12; Combined training (CT): 12	CO: 8 exercises (chest press, chest cross, shoulder press, pull downs, biceps curl, triceps extensions, sit-ups, leg press). ET: Cycling for 20 min (increased by 5 min every 4 weeks) at 60% of estimated VO_2_peak. CT: CO plus ET	12 weeks	Frequency: 2 d/w Reps: 2 × 8–15 (weeks 1–4); 3 × 8-15 (weeks 5–9); 4 × 8–15 (weeks 10–12) Phase velocity: NA Rest: NA Load: Established nRM Progression: Increased when patients exceeded the nRM
Zambom- Ferraresi [40]	Conventional resistance training (CO): 14; Combined training (CT): 14; Control (CG): 8	CO: 6 exercises (leg press, knee extension, knee flexion, chest press, seated row, shoulder press) with weight machines. CT: one d/w of CO and 1 d/w of cycling for 20–35 min at 65–90% of peak heaCO rate (increased each session). CG: No intervention.	12 weeks	Frequency: 2 d/w Reps: 3–4 × 6–12 Phase velocity: NA Rest: NA Load: 50–70% of 1RM Progression: Every 6 weeks (repeating 1RM test)

1RM, one repetition maximum; 15RM, fifteen maximal repetitions; CG, control group; CO, conventional resistance training; CT, combined training; EB, resistance training with elastic bands; ER, resistance training with elastic tubing; ET, endurance training; LIT, low-intensity training; NA, not applicable; nRM, maximum number of repetitions; SEB, single-limb resistance training; TEB, two-limb resistance training.

## Supplementary Materials

The following are available online at https://www.mdpi.com/article/10.3390/healthcare9101397/s1, Table S1: Database search strategy, Table S2: Primary outcome results of the included studies.
