# Supplementary material for: Isolated Resistance Training Programs to Improve Peripheral Muscle Function in Outpatients with Chronic Obstructive Pulmonary Diseases: A Systematic Review
Source: Healthcare (Basel). 2021 Oct 19;9(10):1397. doi: 10.3390/healthcare9101397 (PMC8535539; doi:10.3390/healthcare9101397)
Supplement: Supplementary file 1 [file healthcare-09-01397-s001.zip › healthcare-1392053-supplementary.pdf]

Table S1. Database search strategy.

S/n	Database	Search string
1	PubMed	(((((((((((chronic obstructive pulmonary disease[MeSH Terms]) OR (pulmonary emphysema[MeSH Terms])) OR (chronic obstructive airway disease[MeSH Terms])) OR (chronic obstructive lung disease[MeSH Terms])) OR ("copd"[Title/Abstract])) OR ("chronic obstructive pulmonary disease*"[Title/Abstract])) OR ("coad"[Title/Abstract])) OR ("pulmonary emphysema"[Title/Abstract])) OR ("chronic obstructive airway disease*"[Title/Abstract])) OR ("chronic obstructive lung disease*"[Title/Abstract])) AND ((((((((((((((((((((resistance training[MeSH Terms]) OR ("resistance training"[Title/Abstract])) OR (weight lifting[MeSH Terms])) OR ("weight lifting"[Title/Abstract])) OR (human physical conditioning[MeSH Terms])) OR ("physical conditioning"[Title/Abstract])) OR (Exercise Therapy[MeSH Terms])) OR ("exercise therapy"[Title/Abstract]))) OR ("exercise training"[Title/Abstract])) OR ("weight training"[Title/Abstract])) OR ("strength training"[Title/Abstract])) OR ("muscle training"[Title/Abstract])) OR ("machine training"[Title/Abstract])) OR ("heavy training"[Title/Abstract])) OR ("power training"[Title/Abstract])) OR ("high-intensity training"[Title/Abstract])) OR (rehabilitation[MeSH Terms])) OR ("rehabilitation"[Title/Abstract])) OR ("training"[Title/Abstract])) AND ((((((((((((((((((((muscle strength[MeSH Terms]) OR ("muscle strengt*"[Title/Abstract])) OR (muscle fatigue[MeSH Terms])) OR ("muscle fatigue"[Title/Abstract])) OR (muscle weakness[MeSH Terms])) OR ("muscle weakness"[Title/Abstract])) OR (muscle skeletal[MeSH Terms])) OR ("muscle skeletal"[Title/Abstract])) OR (Physical endurance[MeSH Terms])) OR ("physical endurance"[Title/Abstract])) OR ("muscle power"[Title/Abstract])) OR ("muscle endurance"[Title/Abstract]))) OR (muscle strength

		dynamometer[MeSH Terms])) OR ("dynamometer"[Title/Abstract])) OR (Physical Functional Performance[MeSH Terms]) OR ("Physical Functional Performance"[Title/Abstract])) OR (physical examination[MeSH Terms])) OR ("physical examination"[Title/Abstract])) OR ("muscle"[Title/Abstract])) OR ("muscul*"[Title/Abstract])) OR ("muscle force"[Title/Abstract])))
2	CENTRAL	MeSH descriptor: [Pulmonary Disease, Chronic Obstructive] explode all trees OR MeSH descriptor: [Pulmonary Emphysema] explode all trees OR MeSH descriptor: [Pulmonary Disease, Chronic Obstructive] explode all trees OR MeSH descriptor: [Pulmonary Disease, Chronic Obstructive] explode all trees OR ("copd"):ti,ab,kw OR ("coad"):ti,ab,kw OR ("pulmonary emphysema"):ti,ab,kw OR ("chronic obstructive pulmonary disease"):ti,ab,kw OR ("chronic obstructive airway disease"):ti,ab,kw OR ("chronic obstructive lung disease"):ti,ab,kw AND MeSH descriptor: [Resistance Training] explode all trees OR MeSH descriptor: [Weight Lifting] explode all trees OR MeSH descriptor: [Exercise Therapy] explode all trees OR MeSH descriptor: [Physical Conditioning, Human] explode all trees OR MeSH descriptor: [Rehabilitation] explode all trees OR ("resistance training" OR "weight lifting" OR "exercise therapy" OR "physical conditioning" OR "exercise training" OR " weight training" OR "strength training" OR "muscle training" OR "machine training" OR "heavy training" OR "power training" OR "high intensity training" OR "training" OR "rehabilitation"):ti,ab,kw AND MeSH descriptor: [Muscle Strength] explode all trees OR MeSH descriptor: [Muscle Strength Dynamometer] explode all trees OR MeSH descriptor: [Muscle Fatigue] explode all trees OR MeSH descriptor: [Muscle Weakness] explode all trees OR MeSH descriptor: [Muscle, Skeletal] explode all trees OR MeSH descriptor: [Physical Endurance] explode all trees OR MeSH descriptor: [Physical Functional Performance] explode all trees OR MeSH descriptor: [Physical Examination] explode all trees OR

		("muscle strength" OR "muscle strength dynamometer" OR "muscle fatigue" OR "muscle weakness" OR "muscle skeletal" OR "physical endurance" OR "physical examination" OR "physical functional performance" OR "muscle power" OR "muscle force" OR "muscle endurance" OR "muscle" OR "NEXT muscul*"):ti,ab,kw
3	Web of Science	TS=("Pulmonary emphysema" OR "chronic obstructive pulmonary disease*" OR "chronic obstructive lung disease*" OR "chronic obstructive airway disease*" OR copd OR coad) AND TS=("resistance training" OR "weight lifting" OR "exercise therapy" OR "physical conditioning" OR "exercise training" OR " weight training" OR "strength training" OR "muscle training" OR "machine training" OR "heavy training" OR "power training" OR "high intensity training") AND TS=("muscle strength" OR "muscle strength dynamometer" OR "muscle fatigue" OR "muscle weakness" OR "muscle skeletal" OR "physical endurance" OR "physical examination" OR "physical functional performance" OR "muscle power" OR "muscle force" OR "muscle endurance")
4	Embase	'chronic obstructive lung disease'/exp OR 'chronic obstructive lung disease' OR 'lung emphysema'/exp OR 'lung emphysema' OR 'chronic obstructive pulmonary disease':ti,ab,kw OR 'chronic obstructive airway disease':ti,ab,kw OR 'copd':ti,ab,kw OR 'coad':ti,ab,kw AND 'resistance training'/exp OR 'resistance training' OR 'weight lifting'/exp OR 'weight lifting' OR 'weight machine'/exp OR 'weight machine' OR 'muscle contraction'/exp OR 'muscle contraction' OR 'kinesiotherapy'/exp OR 'kinesiotherapy' OR 'exercise'/exp OR 'exercise' OR 'weight training':ti,ab,kw OR 'strength training':ti,ab,kw OR 'muscle training':ti,ab,kw OR 'torque'/exp OR 'torque' OR 'physical performance'/exp OR 'physical performance' OR 'power training':ti,ab,kw OR 'heavy training':ti,ab,kw OR 'high intensity training':ti,ab,kw AND 'muscle strength'/exp OR 'muscle strength' OR 'dynamometer'/exp OR dynamometer OR 'muscle fatigue'/exp OR 'muscle fatigue' OR 'muscle weakness'/exp OR 'muscle weakness' OR 'skeletal

		muscle'/exp OR 'skeletal muscle' OR 'endurance'/exp OR 'endurance' OR 'physical examination':ti,ab,kw OR 'torque'/exp OR 'torque' OR 'physical performance'/exp OR 'physical performance' OR 'muscle power':ti,ab,kw OR 'muscle force':ti,ab,kw OR 'muscle exercise'/exp OR 'muscle exercise' OR 'muscle endurance':ti,ab,kw
5	Scopus	TITLE-ABS-KEY ("chronic obstructive pulmonary disease" OR "Pulmonary emphysema" OR "chronic obstructive lung disease*" OR "chronic obstructive airway disease*" OR "COPD" OR "COAD") AND TITLE-ABS-KEY ("re-sistance training" OR "weight lifting" OR "exercise therapy" OR "physical conditioning" OR "exercise training" OR " weight training" OR "strength training" OR "muscle training" OR "machine training" OR "heavy training" OR "power training" OR "high intensity training") AND TITLE-ABS-KEY ("muscle strength" OR "muscle strength dynamometer" OR "muscle fatigue" OR "muscle weakness" OR "muscle skeletal" OR "physical endurance" OR "physical examination" OR "physical functional performance" OR "muscle power" OR "muscle force" OR "muscle endurance")

Table S2. Primary outcome results of the included studies.

Study	Population characteristics	Mean age, years	Female, %	FEV_1, % of predicted	Outcome measures	Results	
						Within groups	Between groups
Clark[28]	43 COPD outpatients (non-hypoxaemic, age < 65 years)	49 ± 11	42	77 ± 23	Isotonic knee ext[2] (1RM, Kg)	(Not reported)	RT > CG**
					Isotonic knee flex[2] (1RM, Kg)	(Not reported)	(Not reported)
					Isotonic leg press[2] (1RM, Kg)	(Not reported)	(Not reported)
					Isotonic body squat[2] (1RM, Kg)	(Not reported)	(Not reported)
					Isotonic squat calf[2] (1RM, Kg)	(Not reported)	(Not reported)
					Isotonic chest press[2] (1RM, Kg)	(Not reported)	(Not reported)
					Isotonic lat pull[2] (1RM, Kg)	(Not reported)	(Not reported)
					Isotonic arm curl[2] (1RM, Kg)	(Not reported)	(Not reported)
					Isokinetic knee ext PT[3] ($70°·s^{-1}$, Nm)	(Not reported)	(ns)
					Isokinetic triceps PT[3] ($70°·s^{-1}$, Nm)	(Not reported)	(Not reported)
					Isokinetic knee ext endurance[3] (60 s, $70°·s^{-1}$, J)	(Not reported)	RT > CG*
					Isokinetic triceps endurance[3] (60 s, $70°·s^{-1}$, J)	(Not reported)	(Not reported)

Dourado[29]	35 COPD outpatients	63 ± 29	26	59 ± 29	Isotonic knee ext[2] (1RM, Kg)	RT and CT*, LIT (ns)	RT and CT > LIT*
					Isotonic leg press[2] (1RM, Kg)	RT and CT*, LIT (ns)	CT > RT*, CT and RT > LIT*
					Isotonic chest press[6] (1RM, Kg)	RT and CT*, LIT (ns)	RT > LIT*
					Isotonic lat pull[2] (1RM, Kg)	RT and CT*, LIT (ns)	RT and CT > LIT*
					HGS[1] (KgF)	(ns)	(ns)
Freire[41]	48 COPD outpatients (GOLD I-IV)	68 ± 28	35	49 ± 21	Isotonic knee ext[8] (1RM, Kg)	EB*, ER and RT (ns)	(ns)
					Isotonic knee flex[8] (1RM, Kg)	ER and RT*, EB (ns)	(ns)
					Isotonic shoulder abduction[8] (1RM, Kg)	EB*, ER and RT (ns)	(ns)
					Isotonic shoulder flex[8] (1RM, Kg)	(ns)	(ns)
					Isotonic elbow flex[8] (1RM, Kg)	EB*, ER and RT (ns)	(ns)

Hoff[35]	12 COPD outpatients (age between 40 and 70 years, FEV_1 < 60% of predicted)	62 ± 3	33	36 ± 6	Isotonic leg press[2] (1RM, Kg)	RT*, CG (ns)	RT > CG*
					Leg press dynamic PF[4] (N)	(ns)	RT > CG*
					Leg press static PF[4] (N)	RT*, CG (ns)	RT > CG*
					RFD dynamic[4] ($N \cdot s^{-1}$)	RT*, CG (ns)	(ns)
Kongsgaard[30]	13 COPD outpatients	72 ± 2	0	46 ± 4	Isometric knee ext PT[3] (60°, Nm)	RT*, CG (ns)	RT > CG*
					Isokinetic knee ext PT[3] ($60° \cdot s^{-1}$, Nm)	RT*, CG (ns)	RT > CG*
					Isokinetic knee ext PT[3] ($180° \cdot s^{-1}$, Nm)	RT*, CG (ns)	RT > CG*
					Isotonic leg press[2] (5RM, Kg)	RT*, CG (ns)	RT > CG*
					Max leg ext power[5] (W/Kg)	RT*, CG (ns)	RT > CG*
Nyberg[36]	44 COPD outpatients (GOLD II-IV, non-hypoxaemic)	69 ± 5	48	57 ± 13	Isokinetic knee ext PT[3] ($60° \cdot s^{-1}$, Nm)	EB*, CG (ns)	EB > CG*
					Isokinetic knee ext endurance[3] (30 reps, $300° \cdot s^{-1}$, J)	EB*, CG (ns)	EB > CG*
					Isokinetic shoulder flex PT[3] ($60° \cdot s^{-1}$, Nm)	EB*, CG (ns)	(ns)
					Isokinetic shoulder flex endurance[3] (30 reps, $300° \cdot s^{-1}$, J)	EB*, CG (ns)	EB > CG*

Nyberg[42]	33 COPD outpatients (age ≥ 40 years, FEV_1 < 50% of predicted)	66 ± 7	30	39 ± 10	Isokinetic knee ext PT[3] ($60°\cdot s^{-1}$, Nm)	(ns)	(ns)
					Isokinetic knee ext endurance[3] (25 reps, $60°\cdot s^{-1}$, J)	SEB and TEB*	(ns)
					Isokinetic shoulder flex PT[3] ($60°\cdot s^{-1}$, Nm)	(ns)	(ns)
					Isokinetic shoulder flex endurance[3] (25 reps, $60°\cdot s^{-1}$, J)	SEB and TEB*	(ns)
Ortega[31]	47 COPD outpatients	64 ± 26	13	38 ± 15	Isotonic knee flex[2] (1RM, Kg)	RT, ET and CT*	RT and CT > ET**
					Isotonic knee ext[2] (1RM, Kg)	RT, ET and CT*	RT and CT > ET**
					Isotonic lat pull[2] (1RM, Kg)	RT, ET and CT*	RT and CT > ET**
					Isotonic butterfly[6] (1RM, Kg)	RT, ET and CT*	RT and CT > ET** and RT > CT*
					Isotonic shoulder press[6] (1RM, Kg)	RT, ET and CT*	RT and CT > ET**

Ramos[37]	34 COPD outpatients (non-hypoxaemic)	67	29	Not reported	Isometric dominant knee ext MVC[7] (6 s, N)	RT and ER**	(ns)
					Isometric dominant knee flex MVC[7] (6 s, N)	RT and ER**	(ns)
					Isometric dominant shoulder flex MVC[7] (6 s, N)	RT and ER**	(ns)
					Isometric dominant shoulder abd MVC[7] (6 s, N)	RT and ER**	(ns)
					Isometric dominant elbow flex MVC[7] (6 s, N)	RT and ER**	(ns)
Silva[32]	19 COPD outpatients (age ≥ 45 years)	65 ± 9	Not reported	Not reported	Isometric knee ext MVC[7] (6 s, N)	RT and ER*	(ns)
					Isometric knee flex MVC[7] (6 s, N)	RT and ER*	(ns)
					Isometric shoulder abduction MVC[7] (6 s, N)	RT and ER*	(ns)
					Isometric shoulder flex MVC[7] (6 s, N)	RT and ER*	(ns)
					Isometric elbow flex MVC[7] (6 s, N)	RT and ER*	(ns)
Silva[33]	35 moderate to severe COPD outpatients	68 ± 10	Not reported	49 ± 16	Isometric knee ext MVC[8] (8 s, N)	RT and ER*	(ns)
					Isometric elbow flex MVC[8] (8 s, N)	RT and ER*	(ns)

Simpson[38]	28 COPD outpatients	72 ± 5	46	39 ± 20	Isometric knee ext MVC[7] (90°, N)	RT*, CG (ns)	Not reported
					Single knee ext[2] (1RM, Kg)	RT*, CG (ns)	Not reported
					Single leg press[2] (1RM, Kg)	RT*, CG (ns)	Not reported
					Single arm curl[2] (1RM, Kg)	RT*, CG (ns)	Not reported
					HGS[7] (right, Nm)	RT*, CG (ns)	Not reported
Spruit[39]	30 COPD outpatients	63 ± 7	13	41 ± 19	Isometric knee ext PT[3] (60°, Nm)	RT and ET*	ET > RT*
					Isometric knee ext MVC[7] (60°, N)	RT*, ET (ns)	(ns)
					Isometric knee flex MVC[7] (N)	RT and ET*	(ns)
					Isometric shoulder abd MVC[7] (N)	RT*, ET (ns)	(ns)
					Isometric elbow flex MVC[7] (N)	RT and ET*	(ns)
					HGS[7] (right, N)	(ns)	(ns)
Vonbank[34]	36 COPD outpatients	60 ± 7	31	56 ± 16	Isotonic leg press (1RM, kg)	RT and CT*, ET (ns)	NA
					Isotonic chest press (1RM, kg)	RT and CT*, ET (ns)	NA
					Isotonic lat pull (1RM, kg)	RT*, ET and CT*	NA

Zambom-Ferraresi[40]	36 COPD outpatients (GOLD II-III, age 60 to 80 years)	68 ± 31	0	45 ± 22	Isotonic leg press[2] (1RM, Kg)	RT and CT*, CG (ns)	RT and CT > CG*
					Leg press power output[4] (50% of 1RM, W)	CT*, RT and CG (ns)	Not reported
					Isotonic chest press[2] (1RM, Kg)	RT, CT**, CG (ns)	RT and CT > CG*

*P-value < 0.05; **P-value < 0.001; 1RM, one repetition maximum; 5RM, five maximal repetitions; abd, abduction; CG, control group; CO, conventional resistance training; CT, combined training; EB, resistance training with elastic bands; ER, resistance training with elastic tubing; ET, endurance training; ext, extension; flex, flexion; GOLD, Global Initiative for Chronic Obstructive Lung Disease; HGS, handgrip strength; LIT, low-intensity training; MVC, maximal voluntary contraction; ns, not significant; NA, not applicable; PF, peak force; PT, peak torque; RFD, rate of force development; [1]Measured via hydraulic hand-held dynamometer; [2]Measured via weight-lifting machine; [3]Measured via computerized dynamometer; [4]Measured via force platform; [5]Measured via power rig; [6]Measured via free weights; [7]Measured via digital hand-held dynamometer; [8]Measured via electronic dynamometer.
